# Fulminant invasive aspergillosis of the mediastinum in an immunocompetent host: a case report

**DOI:** 10.1186/1752-1947-6-311

**Published:** 2012-09-18

**Authors:** Muhammad Tariq Shakoor, Samia Ayub, Zunaira Ayub, Faisal Mahmood

**Affiliations:** 1 Department of Internal Medicine, St Mary Mercy Hospital, Livonia, MI, USA; 2 Department of Internal Medicine, Mount Auburn Hospital (Harvard Medical School), Cambridge, MA, USA; 3 Fatima Jinnah Medical College, Lahore, Pakistan; 4 Department of Infectious Diseases, Aga Khan University Hospital, Karachi, Pakistan

## Abstract

**Introduction:**

Invasive aspergillosis is a serious complication in immunocompromised patients. It is an opportunistic disease, which predominantly occurs in the lungs, although dissemination to virtually any organ is possible. Invasive aspergillosis in an immunocompetent patient with extension to the mediastinum has rarely been reported. Here, we present the case of a patient with no apparent immunodeficiency state, who presented with *Aspergillus* endocarditis and fulminant invasive aspergillosis with extensive involvement of the mediastinal structures, which ultimately was responsible for her death. To the best of our knowledge, this is the first reported case in the literature on fulminant invasive mediastinal aspergillosis with extension to the pulmonary vasculature and concomitant *Aspergillus* endocarditis in an apparently immunocompetent patient without pre-existing lung disease.

**Case presentation:**

Our patient was a previously healthy 47-year-old Asian woman, who presented to our emergency room with severe progressive shortness of breath of one month’s duration, associated with orthopnea and unstable vital signs.

**Conclusion:**

Invasive aspergillosis has been described in the presence of pulmonary disease, such as chronic obstructive pulmonary disorder, and one case has been reported in a patient without preexistent disease, but none of these have been fatal. Our case is therefore the first reported case of its kind. Our case shows that fulminant aspergillosis can occur in an immunocompetent host and can be fatal. We conclude that invasive aspergillosis should not be excluded from the differential diagnosis on the basis of immunocompetency.

## Introduction

Invasive aspergillosis is a serious complication in immunocompromised patients. It is an opportunistic disease, which predominantly occurs in the lungs, although dissemination to virtually any organ is possible. Invasive aspergillosis in an immunocompetent patient with extension to the mediastinum has been reported rarely [1-3]. Here, we present the case of a patient with no apparent immunodeficiency with *Aspergillus* endocarditis and fulminant invasive aspergillosis with extensive involvement of the mediastinal structures, which ultimately lead to the death of our patient.

## Case presentation

This is a case history of a previously healthy non-diabetic 47-year-old Asian woman, who presented to out emergency room with severe progressive shortness of breath of one month’s duration, associated with orthopnea. She was well-oriented but tachypneic, bradycardic (30 beats/min) and hypotensive (70/60mmHg). Her initial management included fluid resuscitation, vasopressors (norepinephrine and dopamine) and insertion of a temporary pacemaker for the complete heart block evidenced by her initial electrocardiogram. Her physical examination was unremarkable except for decreased air entry in her right lower chest on auscultation. Her past medical history was significant for an episode of syncope due to complete heart block a year before but she converted back to sinus rhythm without any intervention. At that time, an echocardiogram revealed moderate pulmonary hypertension and mild mitral regurgitation with normal systolic function, and angiography showed normal coronary arteries.

**Figure 1:**
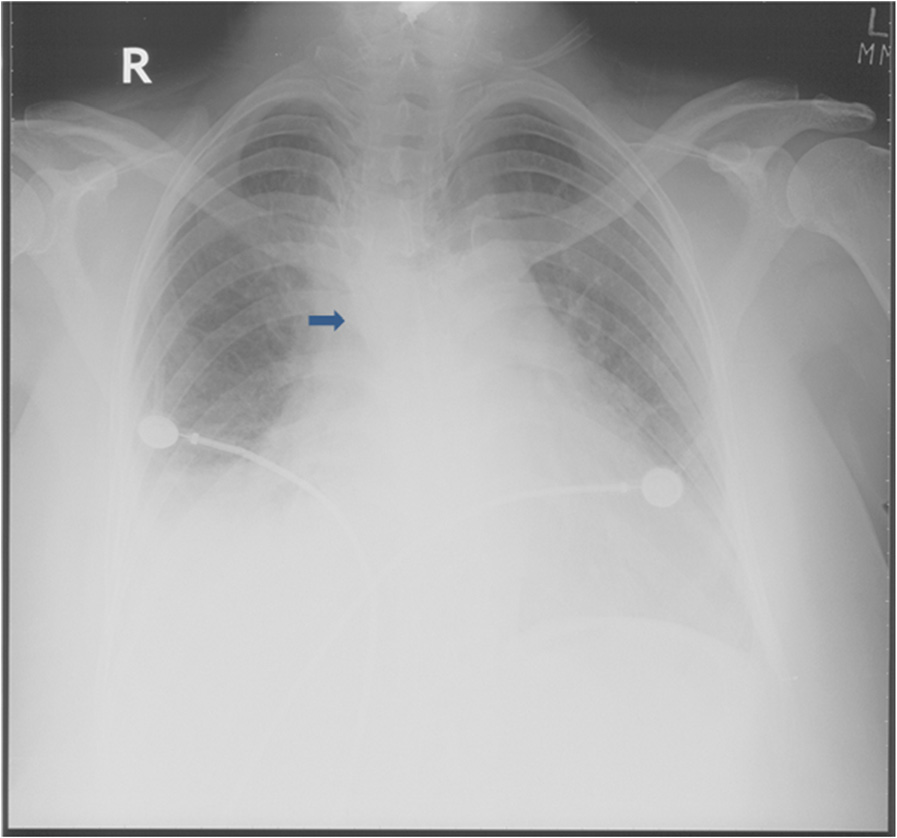
Chest X-ray showing mediastinal widening.

**Figure 2:**
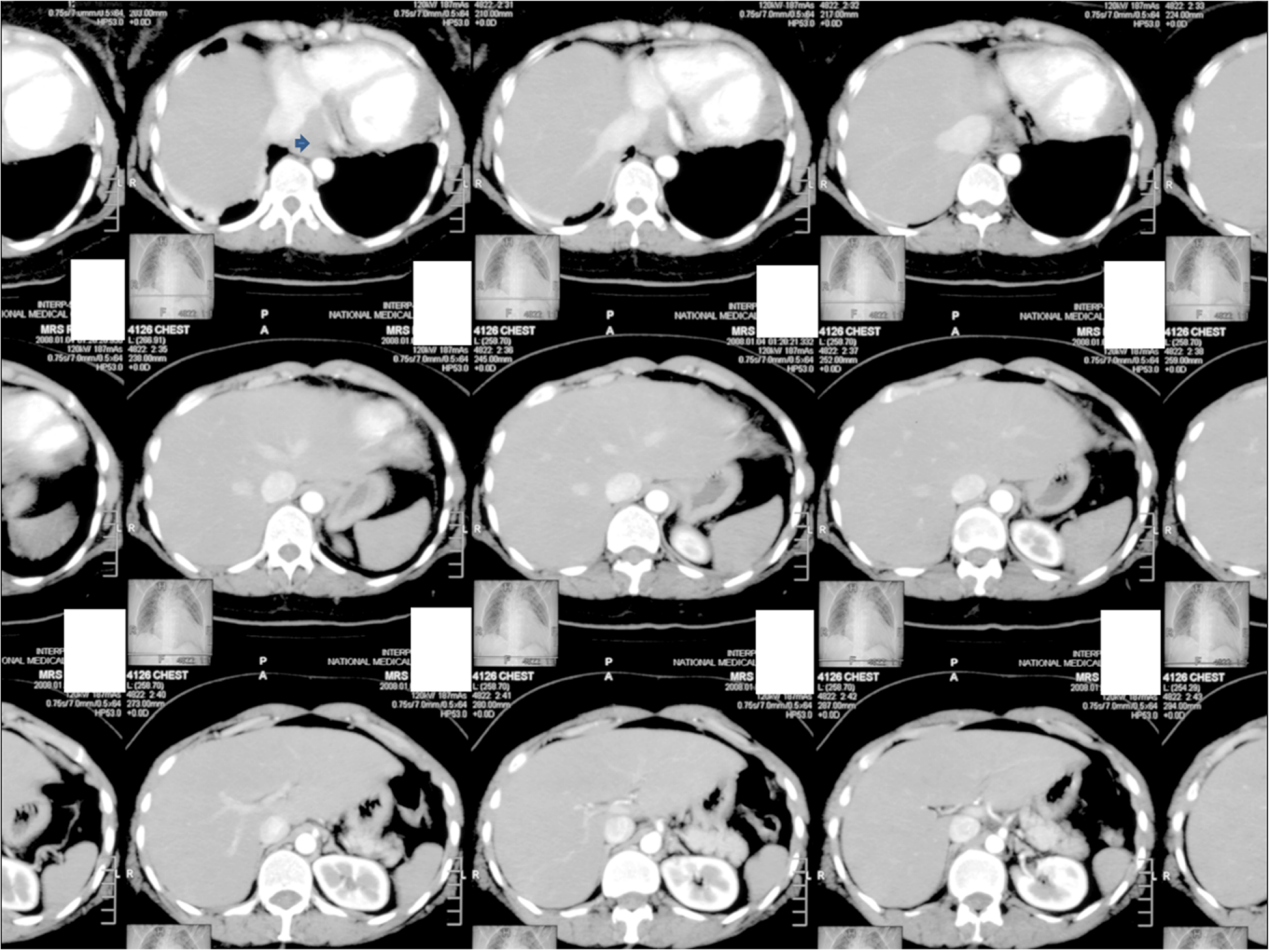
Chest computed tomography showing a right hilar mass involving mediastinal structures.

**Figure 3:**
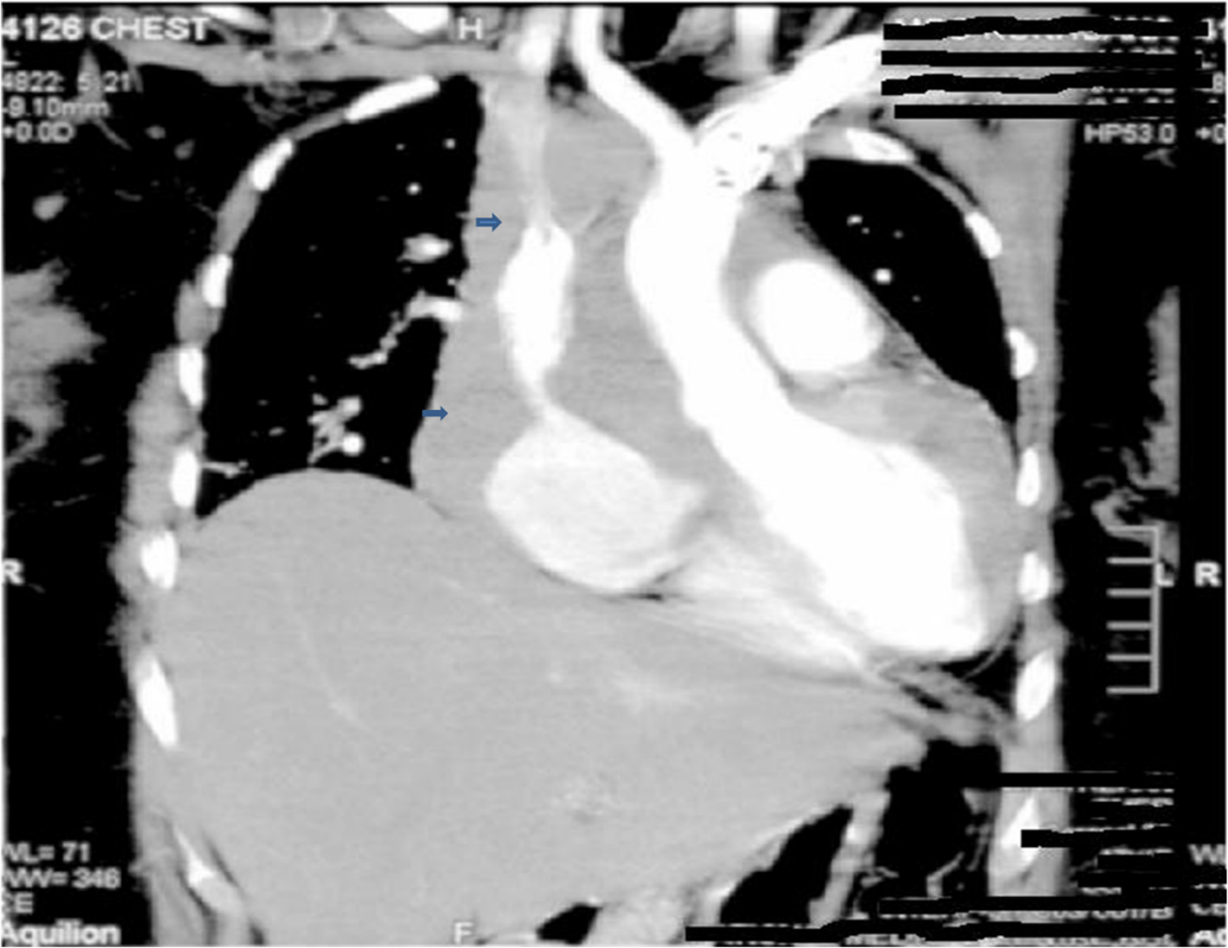
Chest computed tomography showing a right hilar mass involving mediastinal structures.

Her initial laboratory investigations were normal except for marked leukocytosis (19.9 cells/mm3 with 70% polymorpholeukocytes, 27% lymphocytes, 1% eosinophils 2% monocytes) and an elevated level of lactate dehydrogenase (1240IU/L). She was started on broad-spectrum antibiotics (piperacillin and tazobactam (Zosyn®) and vancomycin) after blood cultures were taken. A chest X-ray was significant for mediastinal widening (Figure 1). On further investigation, a computed tomography (CT) scan of her chest revealed a large right-sided mediastinal mass encasing her superior vena cava, ascending aorta, pulmonary vessels and right main bronchus (Figures 2, 3). A presumptive diagnosis of malignancy (likely lymphoma) was made and our cardiothoracic surgery team performed video-assisted thoracoscopic surgery to take a biopsy for further confirmation of the diagnosis. A mass adherent to the posterior surface of her sternum and fixed to her heart was noted. A biopsy of this mass was sent for histopathologic examination and cultures.

Later during the hospital course, she developed a painful red eye, with decreased vision in her right eye. This was evaluated by an ophthalmologist and diagnosed as endophthalmitis. An enzyme-linked immunosorbent assay for human immunodeficiency virus (HIV) was negative. The same day, she had chest pain with ST elevation in the anterior leads on an electrocardiogram (troponin 3.5ng/ml). Repeat echocardiography showed an ejection fraction of 40%, a grossly dilated left atrium and a moderately dilated right atrium. The basal and lateral walls were akinetic and an echogenic density was seen beneath the right septal leaflet. Later that night, her Glasgow Coma Scale score dropped and she required intubation to protect her airway. CT of her head revealed a right frontotemporal region and basal ganglia infarct. She went into cardiac arrest within a few hours; despite advanced cardiac life support, she could not be revived. An autopsy was refused.

**Figure 4:**
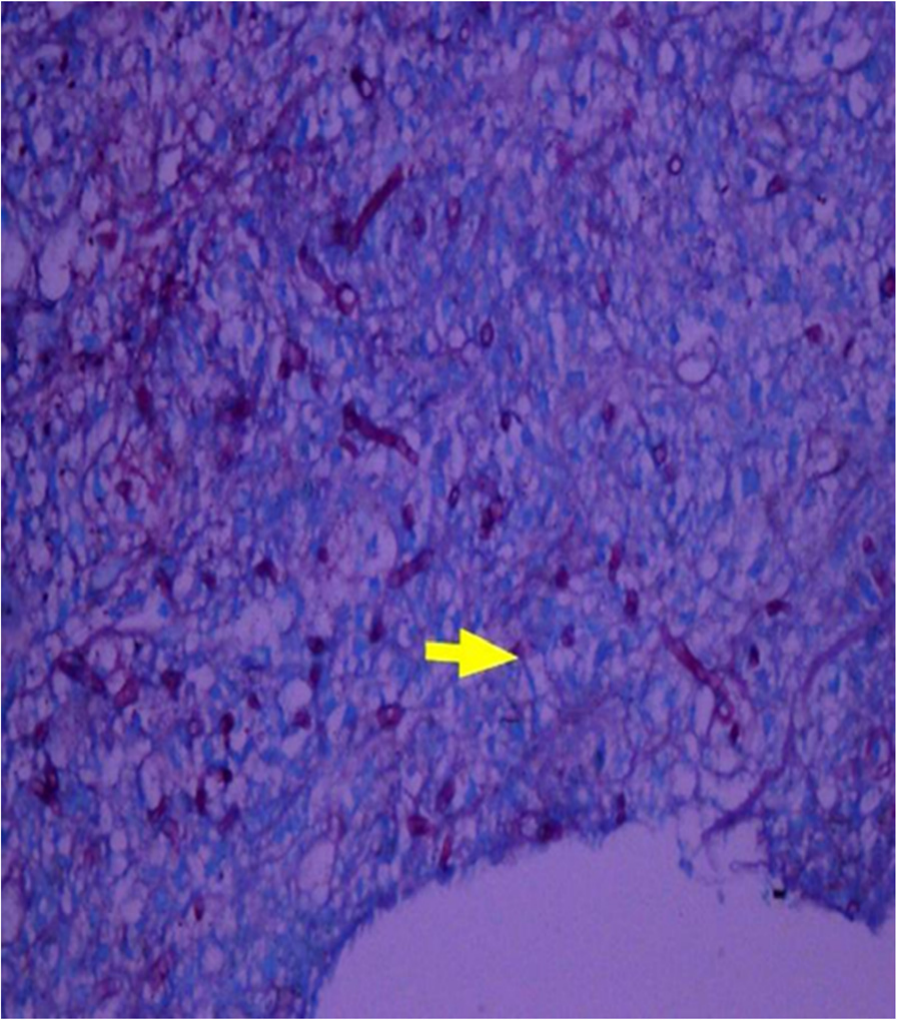
Histology of the specimen showing fungal hyphae, branched at acute angles.

The following day the biopsy report of the mediastinal mass was finalized, and described it as a mass mainly comprising necrotic tissue with abundant fungal spores and hyphae. Hyphae were septated and branched at acute angles (Figure 4). Unfortunately, the mold did not grow in culture. The histopathology report was negative for any underlying malignancy. Nevertheless, these findings led to the diagnosis of a mediastinal mass due to fulminant invasive aspergillosis involving mediastinal structures, and septic emboli as a complication of *Aspergillus* endocarditis.

## Discussion

*Aspergillus fumigatus* is a common commensal of the human airways and causes a broad spectrum of diseases, ranging from hypersensitivity reactions to direct angioinvasion. The major forms of pulmonary aspergillosis range from aspergilloma, with a relatively benign course, to invasive aspergillosis, which has a rapidly progressive course and is highly fatal [4]. Invasive aspergillosis more commonly occurs in immunocompromised patients and, especially in these patients with severe neutropenia, transbronchial invasion can occur. The subsequent invasion of the small pulmonary vessels, with hemorrhage and pulmonary infarct, results in invasive pulmonary aspergillosis, which is responsible for a mortality rate as high as 86%, even with treatment [5]. Rare cases with involvement of the proximal pulmonary arteries, the heart and the aorta have been reported in patients with invasive aspergillosis, but to the best of our knowledge, a case of fulminant invasive mediastinal aspergillosis in an immunocompetent individual has never previously been reported. The past medical history and laboratory work-up (absence of neutropenia, normal blood sugar levels, negative HIV status) and absence of any underlying malignancy (based on the computerized axial tomography of her thorax and abdomen) support the immunocompetent status of our patient.

Reviewing our case in retrospect, our patient presented with a large mediastinal mass on a CT scan, which was found through histopathology to be due to a mold. Although the fungus did not grow in culture, the morphology of acute angled septate hyphae point towards *Aspergillus* species [6]. Regarding the diagnosis of *Aspergillus* endocarditis, we do consider the fact that the echogenic density (most likely a vegetation) in the right septal leaflet of our patient’s valve was not proven through histopathology to be due to a mold, but the course of events are highly supportive of this. Other clinical events that supported this diagnosis were the embolic complications that are well-known in this disease entity. In our patient, the embolic manifestations included endophthalmitis, cerebral infarction and myocardial infarction. A strong association, up to 40%, has been seen between fungal endophthalmitis and fungal endocarditis, which emphasizes the importance of suspecting fungal endocarditis in the setting of such ocular disease [7]. *Aspergillus* endophthalmitis in an immunocompetent person is rarely seen, and only one case has been reported so far in the literature [8]. The rarity of this presentation may have led to the delay in diagnosis.

According to a study conducted over five years and including 152 cases of fungal endocarditis, therapeutic interventions, such as intravascular catheter use and valvular surgery, along with immunosuppressive treatment, were the most common risk factors, although in 3.4% of the total cases, no predisposing factor was identified [9]. Hence, this is possibly a unique case report of invasive mediastinal aspergillosis and native valve fungal endocarditis.

Several factors hamper the timely diagnosis of mold endocarditis. The rarity of the syndrome coupled with negative blood cultures seen in more than 90% of the cases severely compromises the ability to secure an early diagnosis and makes this a diagnostic challenge for clinicians [10].

## Conclusion

To the best of our knowledge, this is the first reported case in the literature of fulminant invasive mediastinal aspergillosis with extension to the pulmonary vasculature and concomitant *Aspergillus* endocarditis in an apparently immunocompetent patient without preexisting lung disease. Rarely, fulminant invasive mediastinal aspergillosis has been described in the presence of pulmonary disease, such as chronic obstructive pulmonary disorder [1,2], and one case has been reported in a patient without preexisting lung disease [3], but none of these reported cases has been fatal. Our case shows that fulminant aspergillosis can occur in an immunocompetent host and can result in the death of the patient. Therefore, invasive aspergillosis should not be excluded from the differential diagnosis on the basis of immunocompetency.

## Consent

Written informed consent was obtained from the patient’s next of kin for publication of this case report and accompanying images. A copy of the written consent is available for review by the Editor-in-Chief of this journal.

## Competing interests

The authors declare that they have no competing interests.

## Authors’ contributions

SA, MTS, ZA and FM supervised the patient directly. MTS, SA and ZA were the major contributors in writing the manuscript. All authors read and approved the final manuscript.
